# Influences of Dispersions’ Shapes and Processing in Magnetic Field on Thermal Conductibility of PDMS–Fe_3_O_4_ Composites

**DOI:** 10.3390/ma14133696

**Published:** 2021-07-01

**Authors:** V. Stancu, A. Galatanu, M. Enculescu, M. Onea, B. Popescu, P. Palade, M. Aradoaie, R. Ciobanu, L. Pintilie

**Affiliations:** 1 National Institute of Materials Physics, Atomistilor 405A, 077125 Magurele, Romania; stancu@infim.ro (V.S.); gala@infim.ro (A.G.); melania.onea@infim.ro (M.O.); bogdan.popescu@infim.ro (B.P.); palade@infim.ro (P.P.); pintilie@infim.ro (L.P.); 2 Faculty of Physics, University of Bucharest, Atomistilor 405, 077125 Magurele, Romania; 3 Department of Electrical Measurements and Materials, Faculty of Electrical Engineering, Technical University Gh. Asachi Iasi, Boulevard Profesor Dimitrie Mangeron 67, 70050 Iasi, Romania; mihaela.aradoaei@academic.tuiasi.ro (M.A.); rciobanu@yahoo.com (R.C.); 4 All Green SRL, 8 G. Cosbuc Street, 700470 Iasi, Romania

**Keywords:** PDMS, magnetite, magnetic field polymerizations, thermal conductivity properties

## Abstract

Composites of magnetite (Fe_3_O_4_) nanoparticles dispersed in a polydimethylsiloxane (PDMS) matrix were prepared by a molding process. Two types of samples were obtained by free polymerization with randomly dispersed particles and by polymerization in an applied magnetic field. The magnetite nanoparticles were obtained from magnetic micrograins of acicular goethite (α-FeOOH) and spherical hematite (α-Fe_2_O_3_), as demonstrated by XRD measurements. The evaluation of morphological and compositional properties of the PDMS:Fe_3_O_4_ composites, performed by SEM and EDX, showed that the magnetic particles were uniformly distributed in the polymer matrix. Addition of magnetic dispersions promotes an increase of thermal conductivity compared with pristine PDMS, while further orienting the powders in a magnetic field during the polymerization process induces a decrease of the thermal conductivity compared with the un-oriented samples. The shape of the magnetic dispersions is an important factor, acicular dispersions providing a higher value for thermal conductivity compared with classic commercial powders with almost spherical shapes.

## 1. Introduction

Composites of magnetic nanoparticles randomly dispersed in a polymer matrix are promising materials widely applied in many engineering areas for magnetic separation, catalysis, MRI contrast agents, or electromagnetic shielding [1,2,3,4,5]. Polydimethylsiloxane (PDMS) is a suitable polymer for embedded composite materials that combine the properties of the matrix and the nanoparticles [6,7,8].

In the early stage of electromagnetic actuator evolution, the actuator was designed by incorporating a bulky permanent magnet placed on the top of the deforming membrane [9]. At the beginning, thin silicon membrane was used as actuator membrane [10,11]. However, silicon is a fragile material with low flexibility and low fracture [11], and for this reason, a lot of research has been done to solve the MEMS problems such as membrane rupture [12]. One way to solve the problem is to use polymer as membrane. In this context, polydimethylsiloxane (PDMS) was demonstrated to be a suitable polymer for composite materials, and has been attracting a large interest in the field of electromechanical actuators, force sensors, piezoelectric generators, and other stretchable electronics [13,14] due to its high flexibility. The final properties of these composites depend upon various parameters, such as size of particles, method of preparation of composite, and dispersion of particles into the polymer matrix [15,16,17]. Spin interactions between magnetic nanoparticles can also influence the final properties of the composites [18,19,20].

Another research direction in the composite materials’ field is the study of anisotropic properties, which can be relevant for material design of particle-reinforced polymer composites for advanced field-assisted additive manufacturing strategies. Anisotropic properties are required for new applications such as flexible electronics [21] or bionic devices [22,23]. Control over particle organization is exercised by enhancing the field strength, which improves particle alignment [24]. Gold and iron oxide nanoparticles were frequently used in biomedical applications [25,26] for magnetic drug targeting, which is a delivery scheme in which the medications and suitable magnetically active components are transported by stable pharmaceutical carriers [27,28].

In the past decade, the combination of the magnetic particles with polymer matrix has been studied as it leads to formation of ferromagnetic polymer composite [29]. Another recent application of polymer–nanoparticles (including magnetic nanoparticles) composite is in thermal pads, designed for a rapid transfer of the heat from electronic devices (e.g., automotive microcontrollers) to the surrounding atmosphere [30,31]. Different strategies were tested to enhance the thermal conductivity of polymer–magnetic nanoparticles composites, such as core-shell structures or other additives in the composite [32,33,34,35,36,37,38,39]. However, the effect of a magnetic field, used to align the magnetic nanoparticles during the polymerization process, on the thermal properties of the composite was less studied [40].

In this work, we examine the thermal conductibility of the composites with two types of magnetite randomly dispersed particles in a PDMS matrix and the influence of applying a magnetic field to modify the particle arrangement on the thermal properties of the composite materials.

## 2. Materials and Methods

The magnetite particles with the acicular structure were obtained starting from the goethite (α-FeOOH). The goethite nanoparticles with 50–100 nm in diameter and a few microns in length were obtained from 5M Fe(NO_3_)_3_ and 1M KOH aqueous solutions, mixed at 70 °C for 48 h. Acicular hematite particles (α-Fe_2_O_3_) were obtained by treating goethite at 400 °C in air for 2 h. When acicular α-Fe_2_O_3_ particles are treat in a reducing gas flow (5% H_2_/Ar) with very high purity 99.999% for 2 h at 300 °C, acicular magnetite (Fe_3_O_4_) is obtained.

A second experimental route to obtain magnetite (Fe_3_O_4_) was to treat the commercial hematite powders with the granular morphology (α-Fe_2_O_3_, Merck, 99.99% purity) in a reducing gas flow (5% H_2_/Ar) for 2 h at 330 °C. Thus, we obtained magnetite grains with dimensions scaling from 100 to 400 nm in size.

**Figure 1:**
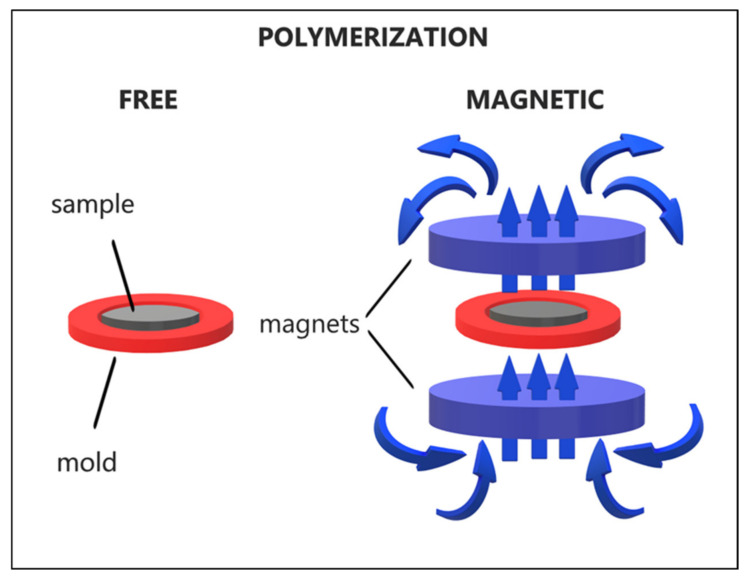
Schematic illustration of the fabrication process.

**Figure 2:**
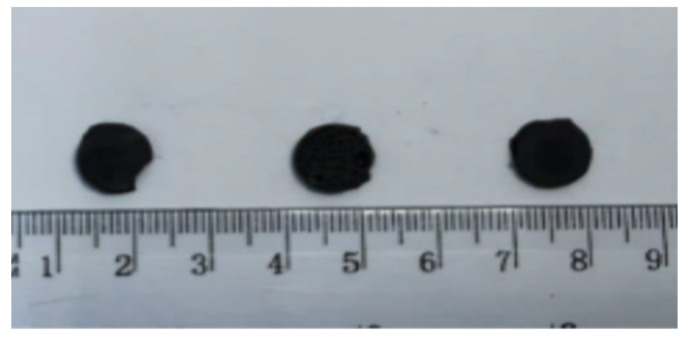
Discs of PDMS:Fe_3_O_4_ composites obtained by free polymerization (left and right) and magnetic polymerizations (middle).

The PDMS base (Sylgard 184, Dow Corning, Midland, MI, USA) and the curing agent with a mass ratio of 10:1 were mixed. Subsequently, the magnetite powders in mass ratio polymer:magnetite of 10:3 were immediately added into the uncured PDMS matrix. After being uniformly mixed, the uncured composite was put into a mold for free polymerization (Figure 1). The oriented sample was obtained by placing the mold into a magnetic field of about 400 mT, as illustrated in Figure 1. Both types of samples were placed in an oven, at 100 °C for 60 min. Thus, we obtained discs of about 10 mm diameter and about 1 mm thickness (Figure 2).

The morphology and elemental compositions of the samples were studied with a Carl Zeiss EVO 50XVP scanning electron microscope (SEM) equipped with Bruker Quantax 200 energy dispersive X-ray spectrometer (EDS) with energy resolution of 129 eV and Peltier cooling, and a field emission scanning electron microscope (FESEM; Gemini 500, Carl Zeiss, AG Germany, Oberkochen, Germany). The crystal structure was analyzed by X-ray diffraction (XRD) using a Bruker D8 Advance equipment (BRUKER-AXS GmbH, Karlsruhe, Germany). Hysteresis loops for acicular magnetite obtained from goethite and for magnetite obtained from commercial hematite were investigated at 300 K using a SQUID magnetometer (MPMS Quantum Design). Thermal conductivity characterizations were performed with Laser Flash Analyzer “Microflash” LFA457 model, Netzsch-Gerätebau GmbH, Germany. All samples were investigated at 25 °C in air, in a transversal configuration, i.e., the heat flow was in the same direction as the magnetic field orientation during the sample processing.

For a good accuracy, the thermal properties results presented were obtained from averaging of 5 measurements for each sample. With the LFA equipment, the thermal diffusivity was directly measured, while the specific heat was obtained by a differential method using a reference material, in this case, a NBS standard alumina sample. The sample and the reference material were exposed to the same amount of laser radiation. To avoid the effects of different reflectance and emissivity of the materials, both materials were covered on both sides with graphite layers having a thickness of a few tens of nanometers and the heating step was analyzed with the same method. Knowing the specific heat of the reference material and having a direct proportionality between the infrared signal step read and the temperature change, the specific heat was obtained as

$$C_{p}^{sample}=\frac{C_{p}^{ref}\times m^{ref}\times \Delta T^{ref}}{m^{sample}\times \Delta T^{sample}}$$

Thermal conductivity, κ, was calculated according to formula κ =α × *C_p_* × ρ, where α is thermal diffusivity, *C_p_* is the specific heat, and ρ is the density of the material. The density was determined by Archimedes method in ethanol at room temperature.

## 3. Results and Discussion

**Figure 3:**
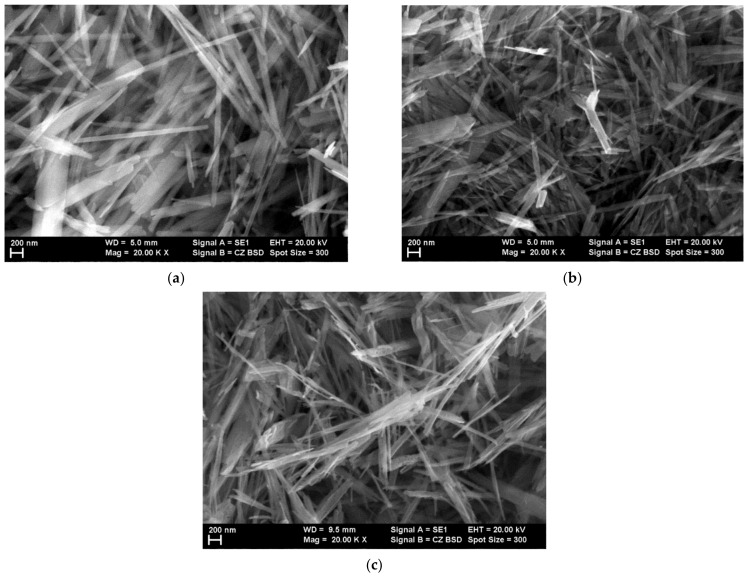

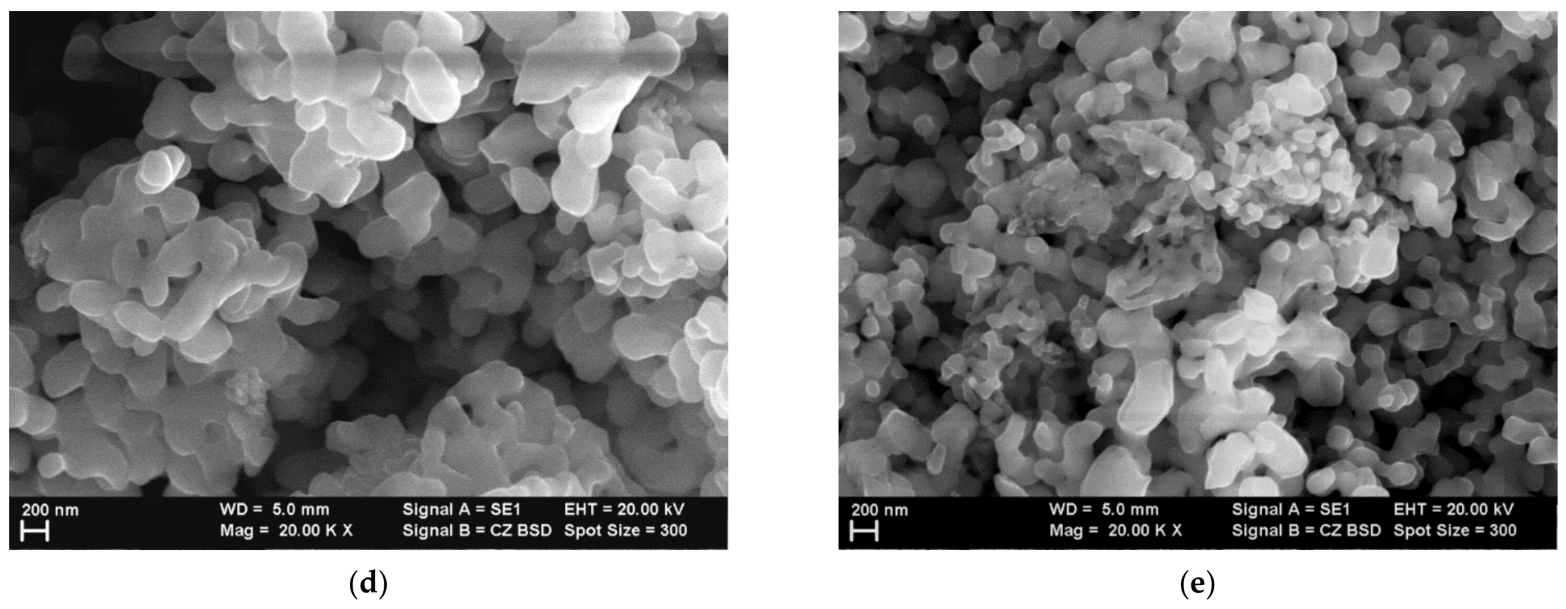
SEM images of (**a**) acicular goethite, (**b**) acicular hematite, (**c**) acicular magnetite, (**d**) commercial hematite, and (**e**) magnetite grains.

The morphologies of the goethite, hematite and magnetite, were evaluated, as presented in Figure 3a–c. It can be observed that the starting materials as well as the magnetite obtained using the first route of fabrication have acicular morphologies. On the other hand, the magnetite obtained by the second route of fabrication has a similar granular morphology as the commercial hematite that was used, as observed in Figure 3d,e.

**Figure 4:**
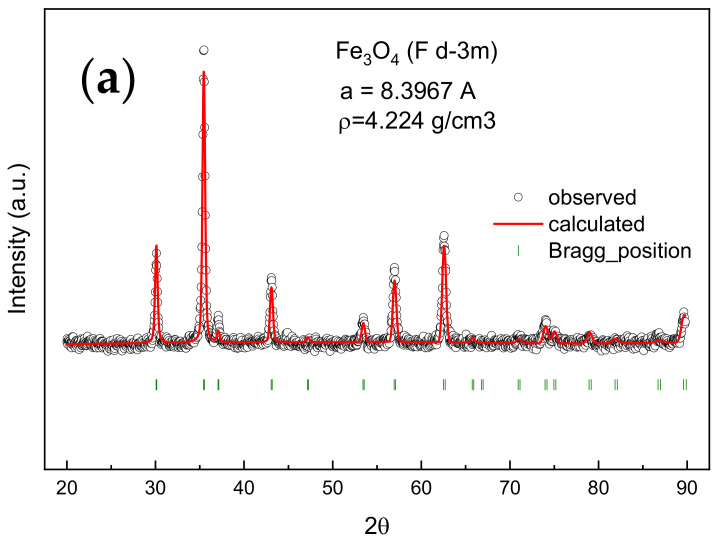

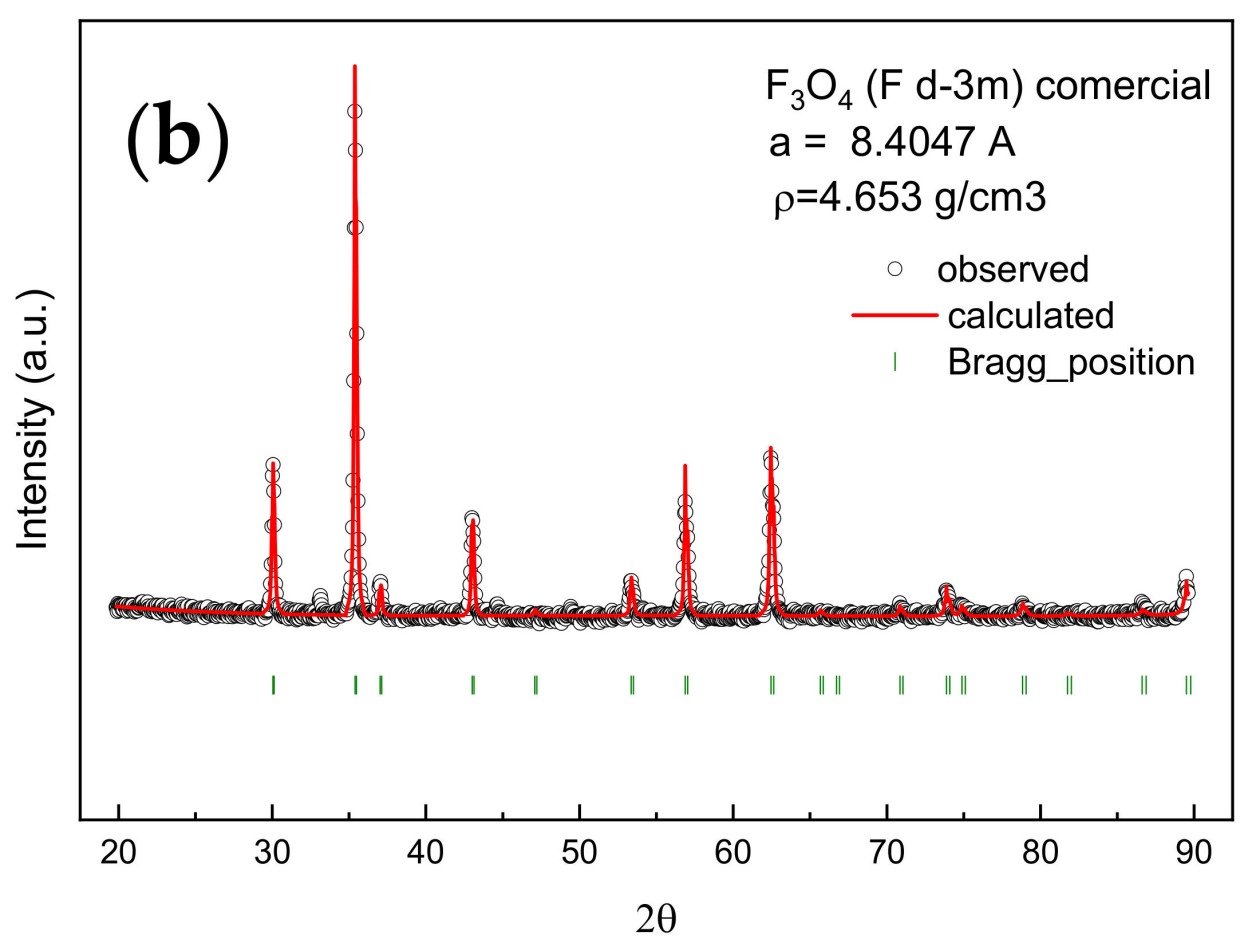
XRD patterns for (**a**) acicular magnetite and (**b**) magnetite obtained from commercial α-Fe_2_O_3_.

The confirmation of obtaining acicular magnetite was achieved by XRD measurements, presented in Figure 4a. The second experimental route of magnetite fabrication was confirmed also by the XRD measurements, as shown in Figure 4b.

**Figure 5:**
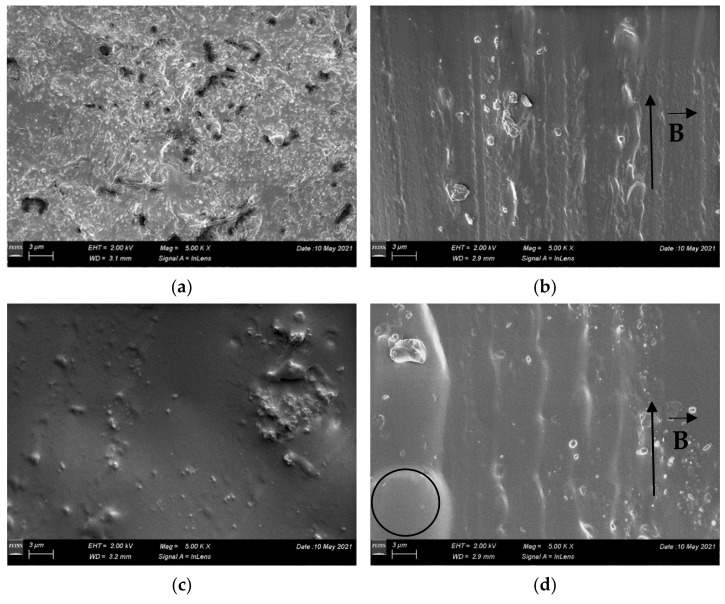
SEM micrographs of acicular PDMS: magnetite composites prepared by (**a**) free polymerization and (**b**) magnetic field polymerization and of spherical PDMS: magnetite composites prepared by (**c**) free polymerization and (**d**) magnetic field polymerization.

The morphology of PDMS:Fe_3_O_4_ composites fabricated using magnetite powders was evidenced by SEM images, as shown in Figure 5. The samples obtained by free polymerization display a good packaging structure, with a homogeneous distribution of the magnetite grains in the PDMS matrix and very small pores (Figure 5a,c). For the samples produced using the magnetic polymerization, the magnetic powder presents an alignment on the direction of the field lines (Figure 5b,d).

In spite of similar composition, the samples exhibit different densities. Moreover, for the sample prepared using the commercial powder of magnetite polymerized in magnetic field, the density is lower than the value obtained for the pristine PDMS. This can be related to the presence of a considerable amount of closed porosity, as indicated in Figure 5d by the circle.

**Figure 6:**
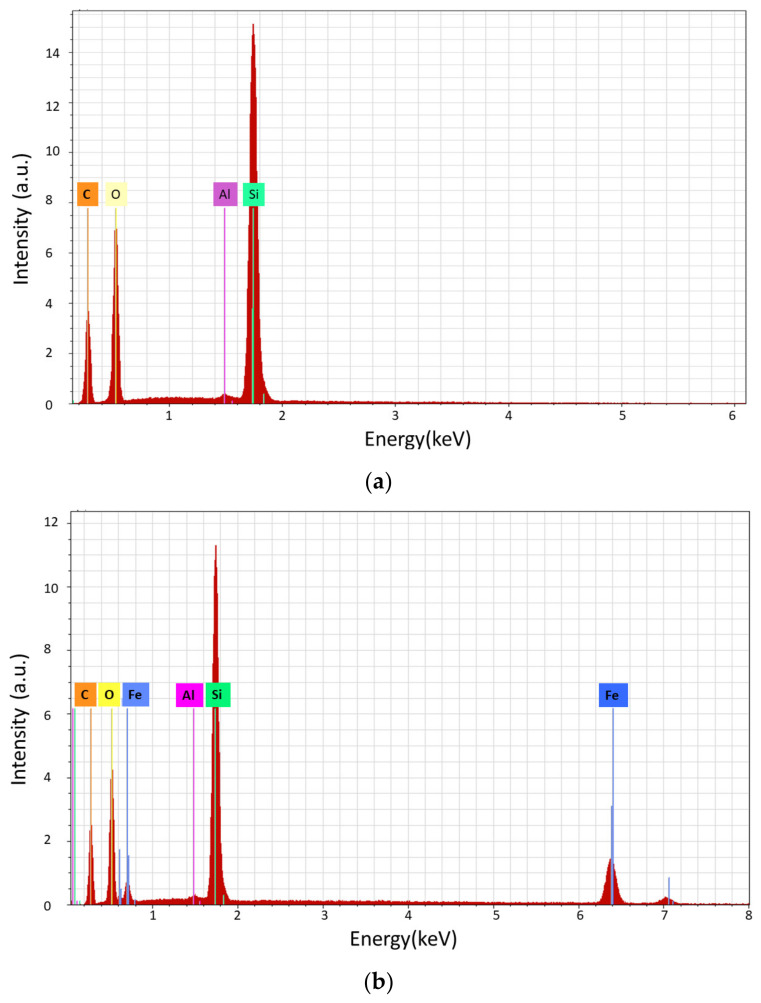
EDX spectra of (**a**) PDMS matrix and (**b**) PDMS: magnetite composites.

The XRD measurements are in a good agreement with compositional EDS analyses (Figure 6). The characteristic bands of the elements from the polymer base composition (C, O, and Si) appear in all spectra and additionally, when magnetite is dispersed in polymer, the very intense Fe bands are evidenced.

**Figure 7:**
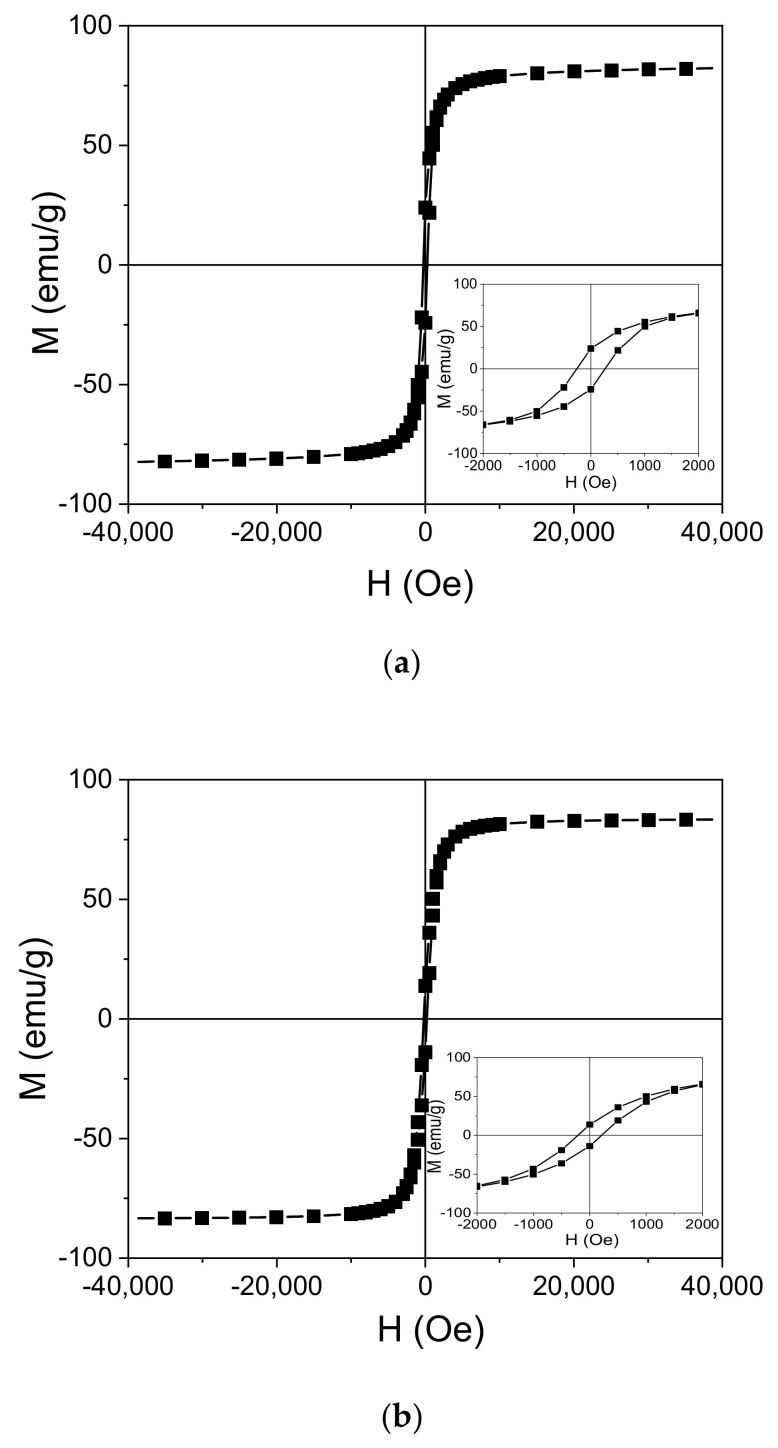
Hysteresis loops measured at 300 K for acicular magnetite obtained from goethite (**a**) and magnetite obtained from commercial hematite (**b**). The insets show the central part of the figures with higher magnification.

In order to assess the magnetic properties of the magnetite powders used to fabricate the PDMS:Fe_3_O_4_ composites, powders were evaluated. Hysteresis loops at 300 K for acicular magnetite obtained from goethite provide the following values: magnetization at saturation (M_s_) of 83.8 emu/g, coercive field of 265 Oe, and remanence M_r_/M_5_ = 29%, where M_r_ is remanent magnetization (Figure 7a). Hysteresis loops at 300 K for magnetite obtained from commercial hematite provide magnetization at saturation (M_s_) of 83.95 emu/g, coercive field of 210 Oe, and remanence M_r_/M_5_ = 17% (Figure 7b).

These values of magnetization at saturation (M_s_) clearly indicate the presence of magnetite. Both hematite and goethite have much smaller M_s_ values, below 5 emu/g.

The high values of M_s_ obtained both for acicular magnetite obtained from goethite and for magnetite obtained from commercial hematite show well-formed magnetite grains with ferrimagnetic (not superparamagnetic) behavior, taking into consideration the significant values of remanence and coercive field. Additionally, both remanence and coercive field are significantly higher for acicular magnetite obtained from goethite than for magnetite obtained from commercial hematite, proving the shape anisotropy due to the acicular shape of the magnetite obtained from goethite.

**Figure 8:**
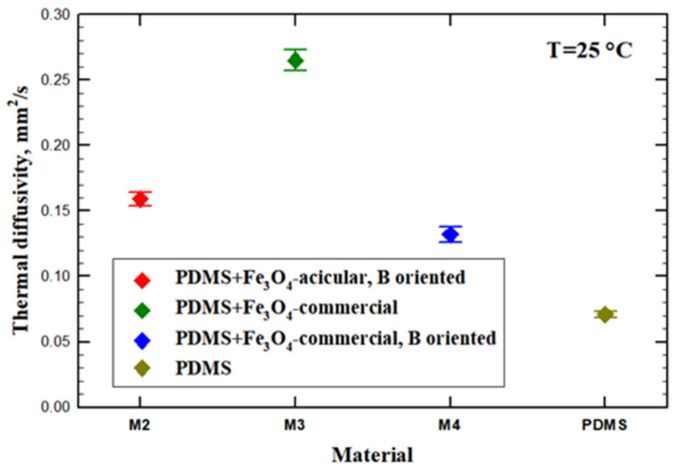
Thermal diffusivity of PDMS: magnetite composites. The points represent the mean values of five measurements, while the horizontal lines are the corresponding error bars.

**Figure 9:**
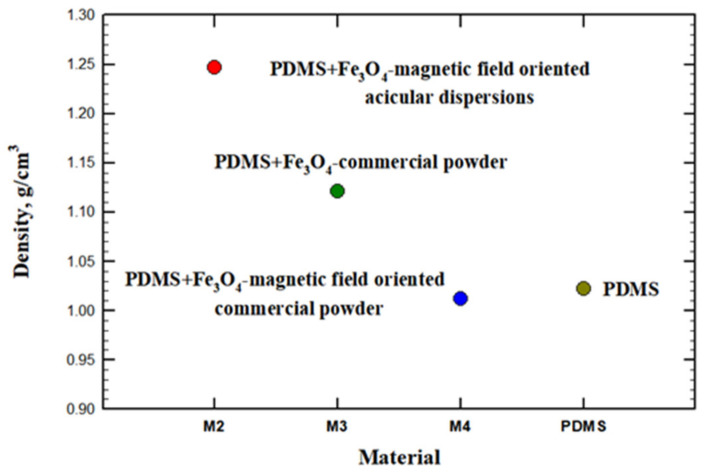
Density of PDMS: magnetite composites determined by Archimedes method.

The thermal diffusivity for the investigated materials is plotted in Figure 8. The diffusivity is a direct measure of the thermal inertia of the material. A higher value indicates a faster spread of the heat in the material. Not surprisingly, the higher value is obtained for the sample produced without an applied magnetic field. Since the powder is magnetic (its Curie temperature being at around 580 °C) the dispersed particles tend to spontaneously agglomerate inside the polymer into clusters, which can eventually create magnetite bridges to transport the heat. The thermal diffusivity of magnetite can be deduced from thermal conductivity values measured in [41] and specific heat data from [42] to be between 0.6 and 0.8 mm^2^/s, that is an order of magnitude higher than the value of PDMS. Thus, contiguity of random patterns of magnetite can provide shortcuts for the heat flux across the sample. Applying a magnetic field creates a more regular pattern for the dispersed powders, which are distributed along the magnetic flux lines, in this case, in the direction parallel to the heat flow. Thus, a decrease of thermal diffusivity is observed for the sample with oriented powders at about half of the value obtained for the sample with unaligned dispersions. It has to be mentioned that using acicular dispersions instead of the commercial powder results in a small increase in thermal diffusivity of that sample compared to the sample with spherical dispersions. A possible explanation might be connected to a better alignment of the shaped magnetite, which could also be concluded for the higher density of this sample (Figure 9), exhibiting a strongly reduced porosity.

**Figure 10:**
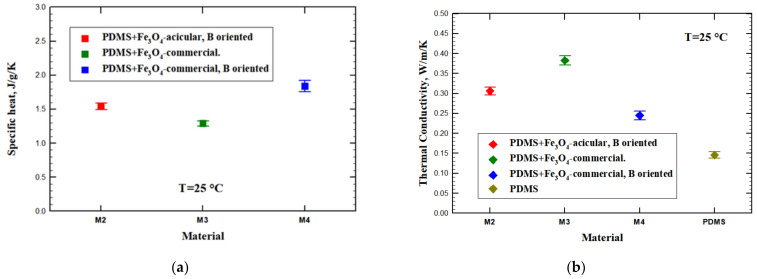
(**a**) Specific heat and (**b**) thermal conductivity of PDMS:magnetite composites. Each point represents the mean value of five measurements, while the horizontal lines are the corresponding error bars.

The specific heat values of the investigated samples are depicted in Figure 10a. From a theoretical point of view, the specific heat of a composite material follows the direct mixture rule, i.e., is the pondered sum of the constituents’ specific heat. The PDMS specific heat value was measured by the LFA differential method on a sample without dispersions, while for magnetite we used the values reported in the literature. The deviations observed from the direct mixing rule and among samples can be related to the presence of porosity. Assuming a 0.65 J/g/K specific heat value at room temperature in the case of magnetite [42] and about 1.87 J/g/K specific heat value for PDMS, the expected composite specific heat should be around 1.59 J/g/K. As it can be seen from Figure 10a, the measured values are close to the theoretical value.

Using the measured values for thermal diffusivity, specific heat, and density, the thermal conductivity was calculated, the results being plotted in Figure 10b. The thermal conductivity values reproduce the trend observed in thermal diffusivity; however, here, the higher density of the sample with acicular dispersions oriented in magnetic field results in an enhancement of the thermal conductivity value, while for the corresponding sample with spherical aligned dispersions, the porosity plays an opposite role reducing the thermal conductivity.

In the sample with unaligned spherical dispersions, self-agglomeration and clustering of the magnetic particles is the most likely explanation for the strongest enhancement of thermal conductivity values observed in this study. In fact, a similar effect was observed also in nanofluids containing even smaller nanometrical particles of Fe_3_O_4_ particles [43].

The presence of magnetic dispersions in the polymer affects the thermal conductivity of the material, increasing its value up to a factor reaching 2.5 times. The unoriented magnetic dispersions give the highest enhancement of the thermal conductivity, but at the cost of a lower density. Thus, the spontaneous agglomeration of magnetic dispersions produces clusters that contribute to an increase of thermal conductivity but also form the solidifying PDMS matrix with many pores. On the other hand, applying a magnetic field to orient the magnetic dispersions has different effects on thermal conductivity, depending also on the shape of dispersions. Acicular dispersions can be better aligned and help produce a denser matrix, while spherical dispersions produce more voids in the PDMS matrix, resulting in a lower density. As a consequence, the composite with spherical dispersions aligned in the field has also a lower thermal conductivity due to the lower density.

## 4. Conclusions

We demonstrated how thermal conductivity of composite materials can be influenced by the orientation of the magnetite added into the polymer matrix. Using the experimental route presented in this study, it is possible to achieve nano-structured anisotropic conductive films with good application in microelectronics. Un-oriented magnetite dispersions in the PDMS matrix are able to increase the thermal conductivity with a factor of 2.5 compared to the pristine PDMS film, but also produce a substantial porosity. Orientation of the included dispersions using a magnetic field during the sample polymerization results in slightly lower thermal conductivity values (still about 2 times higher than the value of the bare PDMS in the case of acicular dispersions). For oriented dispersions, in the case of acicular-shaped magnetite, the thermal conductivity is higher than in the case of spherical magnetite and also the acicular dispersions promote a strongly reduced porosity, almost annihilated, resulting in a more compact and homogenous sample.

## Data Availability

Data is contained within the article.
